# Interventioneller Mitralklappenersatz: Indikationen, Möglichkeiten und Technik sowie wichtige Aspekte für die perioperative Betreuung

**DOI:** 10.1007/s00063-022-00907-7

**Published:** 2022-03-18

**Authors:** Martin Andreas, Markus Mach, Anna Bartunek, Georg Goliasch, Jörg Kellermair, Michael Grund, Paul Simon, Ilinca Damian, Tillmann Kerbel, Andreas Zierer

**Affiliations:** 1 Universitätsklinik für Herzchirurgie, Medizinische Universität Wien, Währinger Gürtel 18–20, 1090 Wien, Österreich; 2 Abteilung für Herz‑, Thorax- und Gefäßchirurgie, Universitätsklinik für Anästhesie, Allgemeine Intensivmedizin und Schmerztherapie, Medizinische Universität Wien, Wien, Österreich; 3 Abteilung für Kardiologie, Universitätsklinik für Innere Medizin II, Medizinische Universität Wien, Wien, Österreich; 4 Klinik für Kardiologie und internistische Intensivmedizin, Kepler Universitätsklinikum, Linz, Österreich; 5 Universitätsklinik für Herz‑, Gefäß- und Thoraxchirurgie, Kepler Universitätsklinikum, Linz, Österreich

**Keywords:** Mitralklappe, Herzkatheter, Mitralklappeninsuffizienz, Herzklappenprothesen, Mitralklappenanuloplastik, Mitral valve, Cardiac catheter, Heart valve prosthesis, Mitral valve insufficiency, Mitral valve annuloplasty

## Abstract

Die interventionelle Therapie der Mitralklappe stellt im Gegensatz zur Aortenklappenintervention eine größere Herausforderung dar. Die Therapie mittels Mitra-Clip zur Rekonstruktion der Mitralklappe wurde erstmals im Jahr 2003 durchgeführt und ist seit dem Jahr 2008 in Europa zugelassen. Die erste Implantation der einzig zugelassenen interventionellen Mitralklappe erfolgte im Jahr 2014, die Zulassung in Europa erfolge im Jahr 2020. Es befinden sich zahlreiche andere Klappensysteme in klinischer Prüfung und werden das Portfolio wahrscheinlich erweitern. In diesem Artikel werden die Indikation für diese neue Therapieform, die technische Umsetzung sowie die spezifischen Herausforderungen für die perioperative Betreuung besprochen.

**Video online:**

Die Online-Version dieses Beitrags (10.1007/s00063-022-00907-7) enthält ergänzendes Videomaterial.

## Abstract (en)

Transcatheter therapy of the mitral valve is more challenging compared to the aortic valve. Interventional therapy with the Mitra-Clip system, mimicking the surgical edge-to-edge repair, was first introduced in 2003 and received the CE mark in 2008. The first implantation of the Tendyne system, which is currently the only commercially available system for mitral valve replacement, was performed in 2014, and routine clinical use was approved in 2020. Several new valve platforms are in clinical development and will expand the treatment portfolio. This article will discuss the clinical indications, technical challenges and specific requirements for perioperative management.

## Hintergrund

Die interventionelle Behandlung der Mitralklappe stellt eine besondere technische Herausforderung dar. Das steht im Gegensatz zur Behandlung der Aortenklappenstenose, die bereits seit 2 Jahrzehnten über kathetergestützte Therapien möglich ist und erst kürzlich eine erweiterte Indikationsstellung in den europäischen Leitlinien erhalten hat [3]. Die Ursache liegt in der variablen Anatomie der Mitralklappe, der komplexen Funktion sowie in den höheren Drücke, bei denen eine Prothese in stabiler Position gehalten werden muss. Als erste interventionelle Therapie der Mitralklappe wurde daher das Mitra-Clip-System entwickelt. Dieses System ermöglicht die Rekonstruktion der Mitralklappe durch das Einsetzen eines Clips, der das vordere mit dem hinteren Mitralsegel verbindet. Diese Technik wurde ursprünglich vom italienischen Chirurgen Ottavio Alfieri (Mailand) entwickelt und bei Patient*innen mit hohem operativem Risiko chirurgisch eingesetzt. Sie konnte nachfolgend durch ein Clipsystem interventionell repliziert werden, das im Jahr 2003 zum ersten Mal eingesetzt wurde. Fünf Jahre später erhielt das System die Zulassung in Europa und wurde seitdem weltweit über 100.000-mal implantiert.

Es wurde die Möglichkeit des interventionellen Mitralklappenersatzes entwickelt

**Figure 1:**
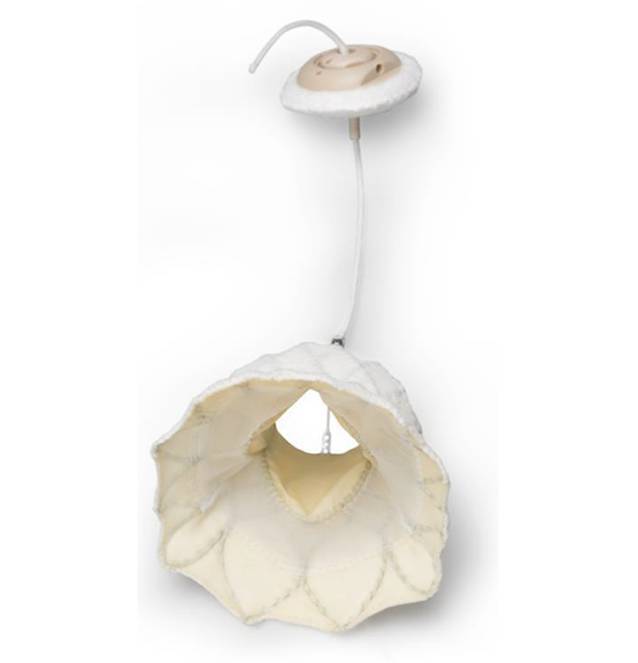

Obwohl der Mitra-Clip große Anwendung findet, gibt es eine große Anzahl an Patient*innen, die für diese Therapie nicht geeignet sind. Auch führt die fehlende Stabilisierung des Mitralanulus zu einem erhöhten Risiko einer erneuten Mitralklappeninsuffizienz. Aus diesem Grund wurde die Möglichkeit des interventionellen Mitralklappenersatzes entwickelt [2]. Das erste System, das die klinische Zulassung erhalten hat, ist die Tendyne®-Klappe (Abbott, North Chicago, IL, USA; Abb. 1; [1]). Sie wurde im Jahr 2014 zum ersten Mal implantiert und konnte Anfang des Jahrs 2020 die klinische Zulassung in Europa erhalten.

## Indikation

Der interventionelle Mitralklappenersatz ist erst kürzlich zugelassen worden, weshalb noch keine große klinische Evidenz vorliegt. Auch Langzeitdaten sind naturgemäß noch nicht vorhanden. Für diese Therapieform kommen Patient*innen mit einem hohen operativen Risiko infrage, um die Risiken einer Herz-Lungen-Maschine oder eines längeren Intensivaufenthalts zu minimieren. Bei Patient*innen, die ein hohes Operationsrisiko haben, aber eine gute Möglichkeit für eine Mitra-Clip-Implantation, ist diese Therapie dem Mitralklappenersatz aus derzeitiger Sicht vorzuziehen, da für die Therapie mit dem Clipsystem aktuell eine breitere klinische Datenlage vorliegt. Derzeit wird zu dieser Fragestellung eine große, internationale Studie durchgeführt (SUMMIT Trial), die beide Therapieformen randomisiert, um damit Aufschlüsse über die Vor- und Nachteile jeder Therapieform zu geben.

Derzeit wird zu dieser Fragestellung eine große, internationale Studie durchgeführt (SUMMIT Trial)

Patient*innen, die grundsätzlich für die Implantation einer kathetergestützten Mitralklappe vorgesehen sind, werden mittels einer 4‑dimensionalen Computertomographie des Herzens vermessen. Wesentlich ist, dass die Vermessungen innerhalb der Spezifikationen für die Klappe sind – der Anulus darf weder zu klein noch zu groß sein – und dass nach der Implantation noch eine ausreichende Öffnung im linksventrikulären Ausflusstrakt zur Verfügung steht. Leider sind etwa 50 % der gescreenten Patient*innen aus anatomischen Gründen derzeit nicht für eine Implantation geeignet. Durch die Etablierung neuer Systeme und stete Verbesserung der Implantationstechnik ist jedoch diesbezüglich in den kommenden Jahren ein deutlicher Fortschritt zu erwarten.

Derzeit werden häufig auch Patient*innen mit dem System behandelt, die eine Verkalkung oder Stenose der Mitralklappe aufweisen und aus diesem Grund nicht für eine andere Therapie infrage kommen. Obwohl diese Patient*innen derzeit „off-label“ behandelt werden, sind die Ergebnisse sehr gut.

## Implantationstechnik (Video 1)

**Figure 2:**
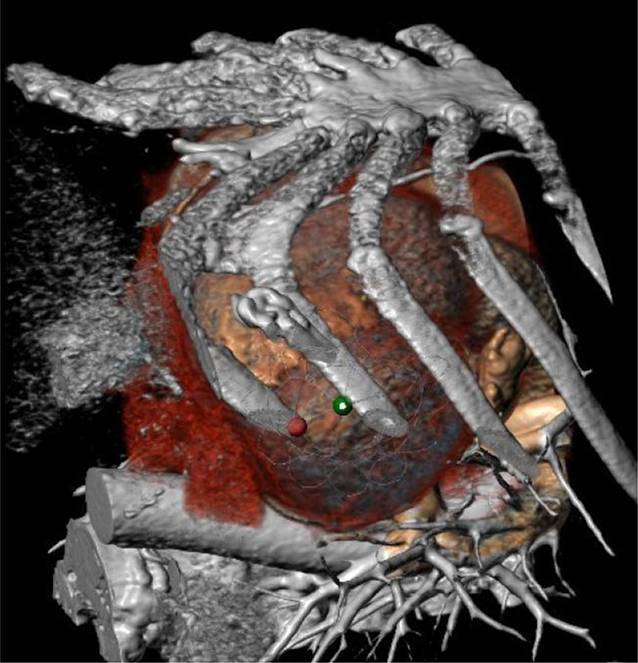

Das in der klinischen Routine angewandte System ist die Tendyne®-Klappe. Dabei handelt es sich um eine transapikale, kathetergestützte Mitralklappe. Der Zugang zur Herzspitze wird über eine linksseitige, anterolaterale Thorakotomie erreicht. Diese ist schon durch die Anwendung der transapikalen „transcatheter aortic valve implantation“ (TAVI) standardisiert und relativ klein im Vergleich zu einer klassischen Thorakotomie. In weiterer Folge wird das Perikard eröffnet und hochgenäht. So kann ein guter Blick auf die Herzspitze erreicht werden. In der präoperativ durchgeführten Computertomographie wird die Stelle des Herzens, die für die ideale Positionierung der Klappe geeignet ist, angezeichnet (Abb. 2). Die korrespondierende Stelle des Herzmuskels wird nun intraoperativ aufgesucht. Anschließend werden 2 dreiecksförmige, pledgetarmierte Tabaksbeutelnähte angebracht, um eine spätere Blutstillung zu ermöglichen. Der Herzmuskel wird punktiert und ein Draht in den linken Vorhof eingebracht. Nach Kontrolle der optimalen Lage wird über diesen Draht das Delivery-System für die Klappe eingebracht. Hervorzuheben ist, dass dieses System einen Durchmesser von 26 French hat.

Das Delivery-System für die Klappe hat einen Durchmesser von 26 French

**Figure 3:**
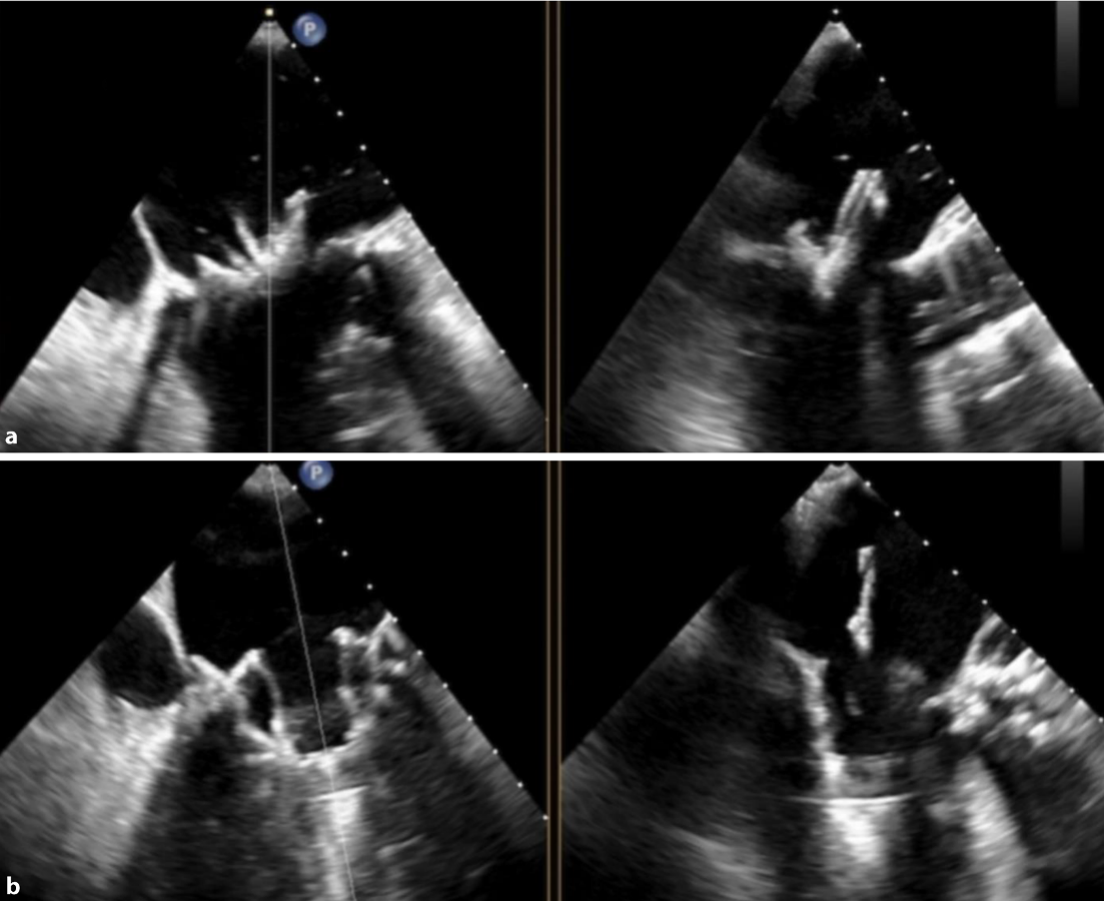

**Figure 4:**
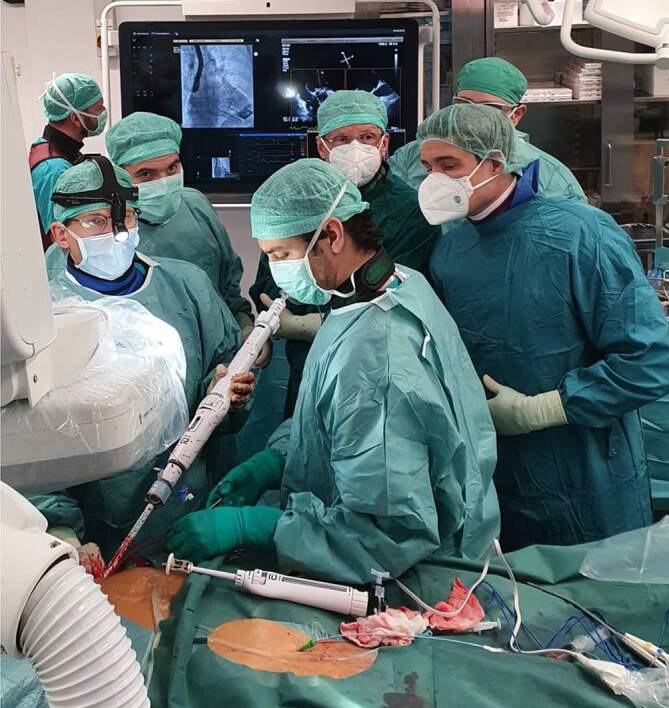

Zur einfacheren Passage wird der Muskel leicht inzidiert. Die Klappe wird nun unter direkter Kontrolle mittels transösophagealer Echokardiographie und Fluoroskopie im Bereich der Mitralklappe positioniert und ausgerichtet (Abb. 3). Nach Erreichen der optimalen Position wird mit dem Öffnen der Klappe begonnen, die schließlich im Anulus der Mitralklappe zu liegen kommt. Die Klappe selbst hat eine D‑Form und ist somit ideal an den Anulus angepasst. Nun wird überprüft, ob die neue Klappe gut arbeitet und ob eine paravalvuläre Undichtigkeit besteht. In weiterer Folge wird auch der Gradient im linksventrikulären Ausflusstrakt hämodynamisch vermessen, um eine Stenose des Ausflusstrakts durch die Klappe auszuschließen. Wenn die optimale Lage bestätigt werden kann, wird nun das Delivery-System entfernt, wobei stets ein Zug auf der Klappe über den Aufhängungsfaden der Klappe gehalten wird (Abb. 4). Nun wird dieser Faden mit einer Scheibe verbunden, die von außen an der Herzspitze herangeführt wird und durch einen vordefinierten Haltedruck die Klappe im Anulus stabilisiert. Der Vorteil dieser Methode ist zum einen eine gute Tonisierung des meist schon geschwächten Ventrikels und zum anderen eine exzellente Hämostase, da sich Blutungen oft durch den Druck der Scheibe selbst tamponieren. Nach erfolgreicher Implantation wird der Thorax mit einer Drainage versorgt und verschlossen.

## Aspekte für die perioperative Betreuung

Der Vorteil der transapikalen Mitralklappenimplantation ist die Vermeidung einer Herz-Lungen-Maschine. Damit wird bei schon geschwächten Herzen oder Patient*innen, die als besonders gebrechlich eingestuft werden, eine schnellere Erholung ermöglicht. Der Eingriff wird in Allgemeinnarkose durchgeführt. Zusätzlich wird während des Eingriffs eine transösophageale Echokardiographie durchgeführt, um die Herzfunktion vor und nach dem Eingriff zu überwachen, eine optimale Position der Klappe zu gewährleisten, die Funktion der Klappe zu beurteilen und eine Stenose des linksventrikulären Ausflusstrakts auszuschließen. Am Ende des Eingriffs wird ein Interkostalblock appliziert, um postoperative Schmerzen zu reduzieren und eine bessere Respiration zu ermöglichen.

Der hämodynamisch kritischste Moment ist das Absetzen der Klappe

Der hämodynamisch kritischste Moment ist das Absetzen der Klappe, da hier praktisch sofort die hochgradige Mitralklappeninsuffizienz komplett eliminiert wird. Das kann für einen bereits geschwächten Ventrikel eine Herzausforderung darstellen. In diesem Fall ist eine Unterstützung mit Inotropika und Volumen möglicherweise indiziert. Wesentlich ist aber gleichzeitig die Etablierung eines entsprechenden systolischen Drucks, um ein Systolic-anterior-movement(SAM)-Phänomen des vorderen Mitralsegels und damit einhergehend eine Obstruktion des linksventrikulären Ausflusstrakts zu verhindern. Hierbei hilft auch die Gabe von Volumen, da dadurch die Füllung des Ventrikels insgesamt besser wird und der Abstand des vorderen Mitralsegels zum Septum zunimmt.

Postoperativ ist meist eine Extubation des Patienten und eine Betreuung im Aufwachraum über Nacht möglich. Damit kann diese Intervention ohne die Nutzung eines Intensivbetts erfolgen, was besonders in der Coronakrise ein Vorteil ist.

## Fazit für die Praxis

- Seit Anfang 2020 steht eine neue Therapie für die Behandlung der schweren Mitralklappeninsuffizienz und in ausgewählten Fällen auch eines kombinierten Vitiums mit Stenosekomponente zur Verfügung.
- Die klinischen Erfahrungen in diesem schwer kranken Patientenkollektiv sind exzellent und lassen auf eine weitere Steigerung dieser Therapieform schließen. Die Etablierung zusätzlicher Systeme in den kommenden Jahren ist zu erwarten.

## Supplementary Information




